# Nanohybridization as a Route to a Water-Friendly Therapeutic Tool for Rescuing Misfolded Proteins

**DOI:** 10.1021/acsnanoscienceau.5c00119

**Published:** 2025-11-07

**Authors:** Mary Bortoluzzi, Aura Cencini, Lavinia Rutigliano, Graziano Rilievo, Alessandro Cecconello, Federica Tonolo, Simone Molinari, Roberta Sacchetto, Marcello Carotti, Dorianna Sandonà, Tiziana Martinello, Juri Ugolotti, Barbara Fresch, Lucio Litti, Fabio Vianello, Massimiliano Magro

**Affiliations:** † Department of Comparative Biomedicine and Food Science, 9308 University of Padua, Viale dell’Università 16, 35020 Legnaro (PD), Italy; ‡ Department of Molecular Medicine, Laboratory affiliated to Istituto Pasteur Italia, Fondazione Cenci Bolognetti, Sapienza, 9311 University of Rome, Viale Regina Elena 291, 00161 Rome, Italy; § Department of Molecular and Translational Medicine, University of Brescia, Viale Europa 11, 25123 Brescia (BS), Italy; ∥ Department of Geosciences, 9308 University of Padua, Via Gradenigo 6, 35131 Padova (PD), Italy; ⊥ Department of Biomedical Sciences, 9308 University of Padua, Via U. Bassi 58/b, 35131 Padova (PD), Italy; # Department of Veterinary Medicine, 9295 University of Bari, Piazza Umberto I 1, 70121 Bari (BA), Italy; g Regional Centre of Advanced Technologies and Materials, Department of Physical Chemistry, Palacky University in Olomouc, Slechtitelu 27, 783 71 Olomouc, Czech Republic; h Department of Chemical Sciences, 9308 University of Padua, Via F. Marzolo 1, 35131 Padova (PD), Italy

**Keywords:** Magnetic nanocarriers, Theragnostic agents, Degenerative disorders, Nanohybrids, Molecular correctors

## Abstract

Misfolded proteins cause several threatening pathologies, ranging from Alzheimer’s disease to cystic fibrosis. Although several protein folding correctors were tested, their delivery is usually inadequate. Here, via a self-assembly wet reaction, colloidal γ-Fe_2_O_3_ was used to immobilize two correctors (C4 and C17) for a cystic fibrosis-associated transmembrane protein. The as-obtained core–shell magnetic nanohybrids were extensively characterized, revealing high drug loading and remarkable chemical stability in water. In addition, a dedicated computational study revealed that the whole organic multilayer is involved in a long-range polarization on the nanoconjugate surface, showing sufficient colloidal stability for its application in cells and in contrast with the cargo’s hydrophobic nature. Experiments conducted in HEK293 cells, expressing a mutated subunit of α-sarcoglycan, showed a positive effect on protein complex recovery. This study represents the first *in* *vitro* example of a multifunctional nanochaperone for the structural recovery of misfolded proteins.

## Graphical abstract

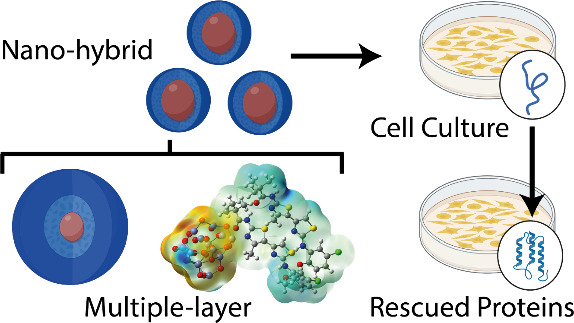

A functional protein acquires a unique 3D conformation via a complicated folding pathway, which is a consequence of the primary amino acid sequence and the local cellular environment. The proper protein conformation is crucial for displaying the correct biological activity; hence, tertiary structure preservation is a relevant cross-cutting topic. The responsiveness of macromolecules to the biological activity of molecular chaperones, which anneal locally unfolded protein regions to provide stability to the overall tertiary structure, is still the object of intense research.^1^

A small error along the folding process could result in misfolded protein structures, which in the worst case can be lethal for the whole cell.^2^ Indeed, diseases such as Alzheimer’s, Parkinson’s, Huntington’s, Creutzfeldt–Jakob, and many other degenerative and neurodegenerative disorders are triggered by protein misfolding.^3^ All of these diseases were found to be related to the aggregation of defective proteins. The oligomeric intermediates induced by misfolding cannot be degraded by common quality control systems in cells, thus becoming potentially toxic.^4,5^ On the other hand, not all misfolded proteins have the propensity to aggregate. In such cases, they are recognized by the cellular quality control machinery, which detects aberrant structural features, such as exposed hydrophobic regions or unstable domains. Rather than accumulating within the cell, the defective protein is targeted for degradation through the ubiquitin-proteasome system or autophagy pathways, thereby preventing its trafficking to the cellular destination.^6^ Examples of this mechanism are class II mutations of the cystic fibrosis transmembrane conductance regulator (CFTR) chloride channel, most notably the ΔF508 variant, responsible for cystic fibrosis (CF). Impaired folding triggers premature degradation via the endoplasmic reticulum-associated degradation (ERAD) pathway, even though the mutant protein may retain partial functional activity, if properly localized. Thus, CF is classified as a loss-of-function disease in which the absence or mislocalization of the protein leads to abnormal ion transport across the cell membrane.

Similarly to cystic fibrosis, there are many other loss-of-function diseases caused by the premature degradation of an otherwise potentially functional protein. Among these, sarcoglycanopathy and Brody disease, which are rare genetic diseases affecting skeletal muscles still without a cure, have been studied for decades.^7–10^ In sarcoglycanopathy, mutations are carried by either alpha-, beta-, gamma-, or delta-sarcoglycan (respectively, α-SG, β-SG, γ-SG, δ-SG), which form a key tetrameric complex that contributes to striated muscle sarcolemma stability.^11^ Mutated sarcoglycans cause severe muscular dystrophy due to the reduction or absence of the SG-complex.^12^ Brody myopathy, characterized by muscle stiffness and impaired relaxation, is caused by mutations in sarco-endoplasmic reticulum calcium ATPase 1 (SERCA1). Missense mutations in α-SG or SERCA1 genes lead to misfolded proteins that, as CFTR type II mutants or sarcoglycan mutants, are prematurely discarded by the cell quality control system, ultimately resulting in lower protein levels.^10^ In an attempt to find a pharmacological therapy for these orphan diseases, small molecules that rescue CFTR type II mutants, known as CFTR correctors, were developed. Among them, the CFTR corrector C17 (*N*-(2-(5-chloro-2-methoxyphenylamino)-4′-methyl-4,5′-bithiazol-2′-yl)­pivalamide) emerged as the most effective in recovering expression and localization of α-SG mutants, first *in vitro* and notably *in vivo*, where the dystrophic phenotype of a mouse model of α-sarcoglycanopathy was significantly ameliorated.^9,13^ Recently, it was proven that C17, along with another bithiazolic derivative, named C4 (*N*-[2-(5-chloro-2-methoxy-phenylamino)-4′-methyl-[4,5′]­bithiazolyl-2′-yl]­benzamide), successfully rescued SERCA1 mutants both *in vitro* and *in vivo*.^9^ The exact mechanism of action of such correctors was not completely clarified, but there is evidence that they can interact and stabilize CFTR proteins, thus acting as a pharmacological chaperone.^14,15^ However, considering their ability to recover structurally different proteins from CFTR, an alternative hypothesis considers C4 and C17 as modulators of the proteostasis network of the cells.^13,16–18^ These findings suggest that these molecules may be used in a potential innovative pharmacological approach to address sarcoglycanopathies and Brody myopathy. Nevertheless, the delivery of CTFR correctors to the exact target site is still a challenging issue due to the hydrophobic nature of the compounds, resulting in a reduced chaperone function.

Life science is constantly fueled by nanotechnology innovations, ranging from biosensors to abiotic material endowed with tailored biological functions.^19–21^ Nanotechnology-based carriers represent a recently introduced option in therapeutic delivery.^22–25^ Nanocarriers are often characterized by multifunctionality, integrating photoluminescence, magnetism, targeting properties, or catalytic activity, and resulting in theragnostic devices.

In the present study, we assembled a novel nanocarrier for CTFR correctors based on in-house produced iron oxide nanoparticles named surface active maghemite nanoparticles (SAMNs). Their superparamagnetism and biocompatibility, along with the potential of improving drug solubility and therapeutic index, as well as for extending drug half-life in the target organ, make them suitable nanocarriers.^26,27^ The two CFTR correctors, C4 and C17, were investigated to provide a proof-of-concept on the feasibility of using CFTR-SAMN hybrids as a protein corrector delivery system. For this goal, a novel protocol for the self-assembly of core–shell nanostructures was developed to obtain a high-yield immobilization of both hydrophobic molecules.

DMSO (dimethyl sulfoxide) can stabilize both organic molecules and the metal oxide nanoparticles in solution. In fact, DMSO is an exceptional solvent for C4 and C17 and, due to its chelating properties, a surface stabilizer for SAMNs.^28^ On this basis, a proper DMSO–water ratio was used to maintain the drugs in solution long enough to generate chemical contact with the naked nanoparticle surface and, at the same time, to thermodynamically drive the system toward the core–shell formation. Therefore, the reaction was investigated at a constant SAMN and drug concentration and a variable DMSO content in water as the solvent (5 to 90% v/v). The optimized binding conditions, in terms of DMSO fraction, were different for the two tested CTFR correctors and resulted in 50% and 20% for C4 and C17, respectively. Assessment of cargo loading was carried out using ultraviolet-visible (UV–Vis) spectroscopy at 324 and 318 nm. Figure 1(a) shows representative spectra for both correctors, where the calculated molar extinction coefficients resulted in ε_324_ = 2.6 × 10^4^ M^–1^ and ε_318_ = 2.8 × 10^4^ M^–1^ for C4 and C17, respectively (calibration curves are reported in Figure S1). The amount of bound organic cargo was quantified by measuring the supernatant concentration after the self-assembly reaction and the nanoparticle magnetic removal. The estimated amount of bound correctors was approximately 150 mg g^–1^ of SAMNs for both C4 and C17. Stability tests confirmed the robustness of the therapeutic cargos, in terms of coating leakage and chemical degradation (for an extensive chemical-physical characterization of SAMN@C4 and SAMN@C17 complexes, see the Supporting Information section).

**Figure 1:**
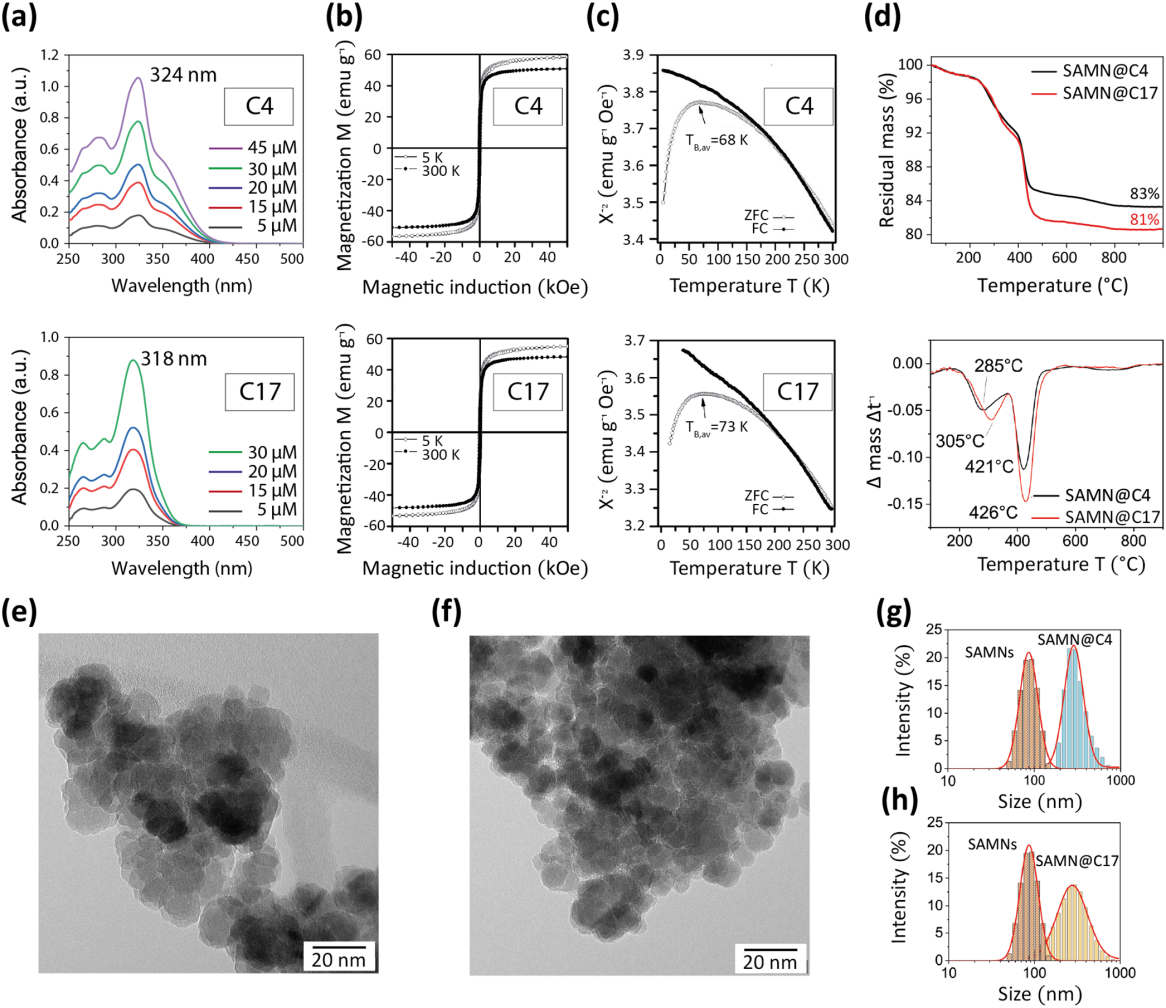
Chemical-physical characterization of SAMN@C4 and SAMN@C17 hybrids. (a) UV–Vis spectra of different concentrations of free (top panel) C4 and (bottom panel) C17 in 50% and 20% DMSO, respectively. (b) Hysteresis loops recorded at 5 and 300 K of (top panel) SAMN@C4 and (bottom panel) SAMN@C17. (c) Bulk magnetization as a function of temperature of SAMN@C4 (top panel) and SAMN@C17 (bottom panel), followed in the field-cooled (FC) and zero-field-cooled (ZFC) modes, and recorded under an applied magnetic field of *B* = 0.1 T. TB represents the blocking temperature. (d) Comparison of the thermogravimetric analysis (TGA) results of SAMN@C4 and SAMN@C17 (top panel) and deconvolution of the TGA curves (bottom panel). Transmission electron microscopy (TEM) images of (e) SAMN@C4 and (f) SAMN@C17. Dynamic light scattering (DLS) measurements of the hydrodynamic volume of (g) SAMN@C4 and SAMNs and (h) SAMN@C17 and SAMNs.

In Figure 1(b) and (c), to further validate the formation of the core–shell hybrids, as well as to quantify the drug loading, the optimized reaction products were analyzed using superconducting quantum interference device (SQUID). Magnetic measurements revealed the contribution of a massive diamagnetic organic phase, very likey ascribable to a high percentage of drug loading. However, the synthetic nanocarriers still displayed very favorable magnetic properties for their use as nuclear magnetic resonance (NMR) contrast agents. In Figure 1(d), thermogravimetric measurements confirm immobilization magnitude to be around hundreds of milligrams per gram of hybrid, representing a satisfactory yield for the presented protocol and, very likely, for application purposes. Furthermore, SAMN@C4 and SAMN@C17 morphological and hydrodynamic features were studied to assess their applicability as drug nanocarriers *in vitro.*

In Figure 1(e) and (f), transmission electron microscopy (TEM) images reveal core–shell structures and the presence of a bulky, less electron-dense phase enveloping the maghemite core. The organic matrix can be attributed to the corrector coating. Its thickness (3 nm), along with thermogravimetric analysis (TGA) and SQUID data, suggests the formation of a drug multilayer, likely due to molecular stacking phenomena. In order to better appreciate the size increase accompanying drug loading, the two nanohybrids were further characterized by measuring their hydrodynamic volumes. While bare SAMNs showed a hydrodynamic diameter of 90 ± 8 nm, the sizes of SAMN@C4 and SAMN@C17 hybrids were 300 ± 20 nm and 330 ± 30 nm, respectively, as reported in Figure 1(g) and (h). The registered 3-fold increase further demonstrates the formation of a massive drug layer around the maghemite core. It is worth mentioning that cell uptake of the SAMN-based nanoconjugate was already observed for nanocarriers in a size range comprising those reported here.^26,27^

Drug immobilization was also monitored by zeta potential, ζ. According to DLS measurements, the ζ of bare SAMNs measured at neutral pH is extremely positive (+39 ± 9 mV) (see Figure S2 for more information). Zeta potentials of nanocarriers upon binding of C4 and C17 were +1.6 ± 0.2 mV and +15 ± 2 mV for SAMN@C4 and SAMN@C17, respectively. It can be assumed that the binding on the nanoparticle surface induced a long-range polarization involving the whole hydrophobic multilayer, possibly explaining the registered nonzero ζ value.

At the physical boundary of the iron oxide nanoparticles, the crystal is interrupted, and therefore, the surface exposes a distribution of uncoordinated iron­(III) sites to the surrounding medium. Ligand binding is therefore thermodynamically spontaneous, as it rescues these reactive dangling bonds.^26,27^ This phenomenon is known as surface reconstruction and is accompanied by a redshift of the nanoparticle UV–Vis absorption spectrum.^29^ In this view, SAMNs represent an elective model, and the redshift effect emerged as a common motif in previous studies, including nanoparticle hybridization with peptides.^26^ To investigate the surface reconstruction phenomenon by C4 and C17, UV–Vis absorption spectra were collected for both hybrids. The integration of C4 and C17 with SAMNs induced an apparently moderate redshift of the absorption maximum of about 10 nm, Figure 2(a), and the appearance of a shoulder at around 493 nm, suggesting that the first organic molecular layer is stabilized through chelation of the bond-deficient iron atoms exposed on the metal oxide surface. To better perceive the magnitude of the optical phenomenon, UV–Vis profiles of the nanohybrids were deconvoluted as described in the Materials and Methods section (Supporting Information). In Figure S3, it is possible to appreciate the similarity of the simulated UV–Vis plots of SAMNs, SAMN@C4, and SAMN@C17 compared to the actual experimental spectra. Upon deconvolution, Figure 2(b,c), two distinctive signals at 462 and 455 nm can be identified as individual features of SAMN@C4 and SAMN@C17, respectively, and can be ascribed to an electron density reorganization upon corrector–surface interaction. Most importantly, this analysis provides preliminary clues on the coordinative nature of the SAMN–corrector interaction.

**Figure 2:**
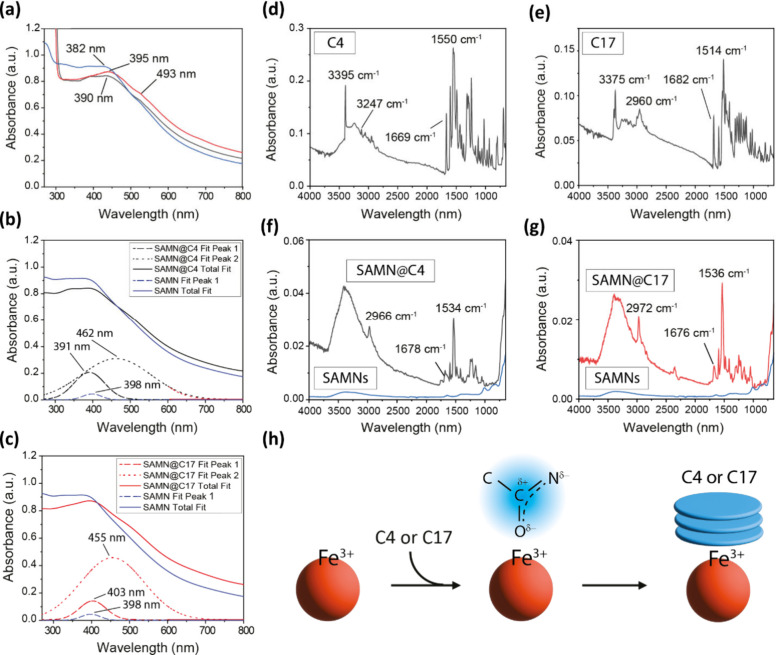
Chemical-physical characterization of the interaction between nanoparticles and correctors through UV–Vis and Fourier-transform infrared (FTIR) spectroscopies. (a) Comparison of bare SAMNs (blue line), SAMN@C4 (black line), and SAMN@C17 (red line) UV–Vis spectra. (b) Deconvolution of the SAMN@C4 UV–Vis spectrum. (c) Deconvolution of the SAMN@C17 UV–Vis spectrum. (d) Infrared spectra of C4; (e) infrared spectra of C17; (f) infrared spectra of SAMN@C4 (black line) and bare SAMNs (blue line); (g) infrared spectra of SAMN@C17 (red line) and bare SAMNs (blue line). (h) Schematic representation of the C4/C17 amide interaction with the SAMN surface and stacking of correctors due to polarization.

To identify the molecular features involved in the coordinative binding of iron­(III), Fourier-transform infrared (FTIR) spectra were collected. These were used to further understand the role of the undercoordinated sites lying on the nanomaterial surface, as well as to detail the effect of cargo nanoimmobilization on its chemical-physical properties. Figure 2(d) and (e) reports infrared analyses of the organic compounds C4 and C17, showing densely crowded signals in the spectral region comprised between 1750 cm^–1^ and 700 cm^–1^, being further complicated by several overlapping phenomena. For simplicity, rather than individual peak attributions of this busy fingerprint region, our attention was focused on the fate of secondary amide vibrations, which are identifiable in both the soluble and immobilized organic molecules (*vide infra*). The vibrational contributions from methoxy, phenyl, amino, and methyl groups, bithiazole rings, and all other molecular constituents were collectively compared to the intensity of the secondary amide vibrational modes. It should be considered that secondary amide C=O stretching and N–H in-plane bending form a diagnostic pair of sharp, intense peaks in the middle of the spectrum. In general, the C=O stretching falls in the 1680 cm^–1^ to 1630 cm^–1^ range, typical of all amides, while its companion peak, the in-plane N–H bending, is normally found from 1570 cm^–1^ to 1515 cm^–1^. These represent a signature of the secondary amide together with the single N–H stretching. In particular, the single N–H stretching is a wide peak of medium intensity, commonly found from 3370 cm^–1^ to 3170 cm^–1^.^30^ In Figure 2(d) and (e), this trio of peaks is highlighted for both free C4 and free C17.

Next, FTIR profiles of SAMN@C4 and SAMN@C17 hybrids were collected (Figure 2(f) and (g), black and red line, respectively). These were characterized by the typical γ-Fe_2_O_3_ features with crystalline Fe–O stretching vibrations located below 800 cm^–1^, compatible with the well-preserved magnetic core.^31^ Moreover, in comparison to bare nanoparticles (Figure 2(f) and (g), with blue lines), the hybrids showed a blooming of signals in the spectral region between 800 cm^–1^ and 1750 cm^–1^, attributed to the surface modifiers. Interestingly, nanoimmobilized C4 and C17 FTIR spectra were nearly superimposable; thus, they will be commented on together.

Upon binding, the vibrational features of the organic phase were significantly reduced in terms of both intensity and number. Thus, besides demonstrating the successful self-assembly reaction, these results point to a strong interaction with maghemite, providing rigidity to the molecules and resulting in a substantial restriction of the vibrational freedom of the molecular groups.

Most notably, among all the features in this spectral region, the amide in-plane N–H bending appeared dominant, towering over other contributions. This in-plane bending is more intense than others because of the conjugation that takes place in amides,^32^ revealing an involvement of the secondary amide in the chelation of undercoordinated iron­(III) sites.^33^ Furthermore, in comparison to the unbound molecules, the amide in-plane N–H bending is characterized by a significant shift upon SAMNs binding, from 1550 cm^–1^ to 1534 cm^–1^ for C4 (Figure 2, panels (d) and (f)) and from 1514 cm^–1^ to 1536 cm^–1^ for C17 (Figure 2, panels (e) and (g)), in line with already reported SAMNs surface modifications.^26^

The occurrence of a chelation phenomenon was further corroborated by observing the binding effect on the secondary amide C=O stretching, which appeared to be dwarfed with respect to the same feature in the parent C4 and C7 FTIR profiles. It is worth considering that the secondary amide displayed an electron delocalization involving nitrogen, carbon, and oxygen atoms due to its partial double-bond character. In this view, the secondary amide group could be considered as a chelating ligand for Fe­(III) sites. This would likely explain the observed remarkable vibrational intensity change. Therefore, although the contact was thermodynamically induced by the solvent, the initial binding respected the typical coordinative nature of the interactions established between the SAMNs and any other surface modifier.

In order to build a molecular view of the interactions between the correctors and the nanoparticle surface, a model of the iron-oxide cluster with Fe_2_O_3_ stoichiometry and its interaction with C4 and C17 were computationally investigated at the quantum-mechanical density functional theory (DFT) level. Studies on the electronic structure of stoichiometric neutral (Fe_2_O_3_)_*n*_ clusters were previously reported at the DFT level up to *n* = 2.^33,34^ Based on this building block, bigger clusters up to *n* = 10 were considered, highlighting a rich landscape of structural isomers and spin configurations.^35–37^ It was found that from *n* = 4 the precise spin configuration has only a minor role in the relative energies of different cluster isomers. To study the local interactions at the SAMN–corrector interface, a stoichiometric cluster with *n* = 7 was adopted to model the iron oxide surface. The bare and ligated model clusters were optimized without symmetry constraints to fully describe the local surface modifications induced by the binding. In order to better appreciate their molecular involvement in the interaction with the inorganic surface, Lewis structures of the therapeutic cargos are reported in Figure 3(a), with the amide functionalities, the main contributor to the interaction, highlighted in red. Furthermore, Figure 3(b) and (c) show the isolated (Fe_2_O_3_)_7_ cluster and the oxide cluster–corrector complexes, respectively. Computational methods and further details on the optimized structures are reported in the Supporting Information (see also Figures S4, S5, and Videos V1, V2, for tridimensional renderings of monomolecular and bimolecular complexes). The model structures were then used to simulate infrared (IR) spectra, reported in Figure 4(a,b), where a reduction in the amide peak intensities is observed upon C17-SAMN complex formation, corroborating the pivotal role of the group in the coordinative anchoring of the surface (see Figure S6 for the analogue SAMN@C4 calculated IR spectra and the SI for a detailed discussion).

**Figure 3:**
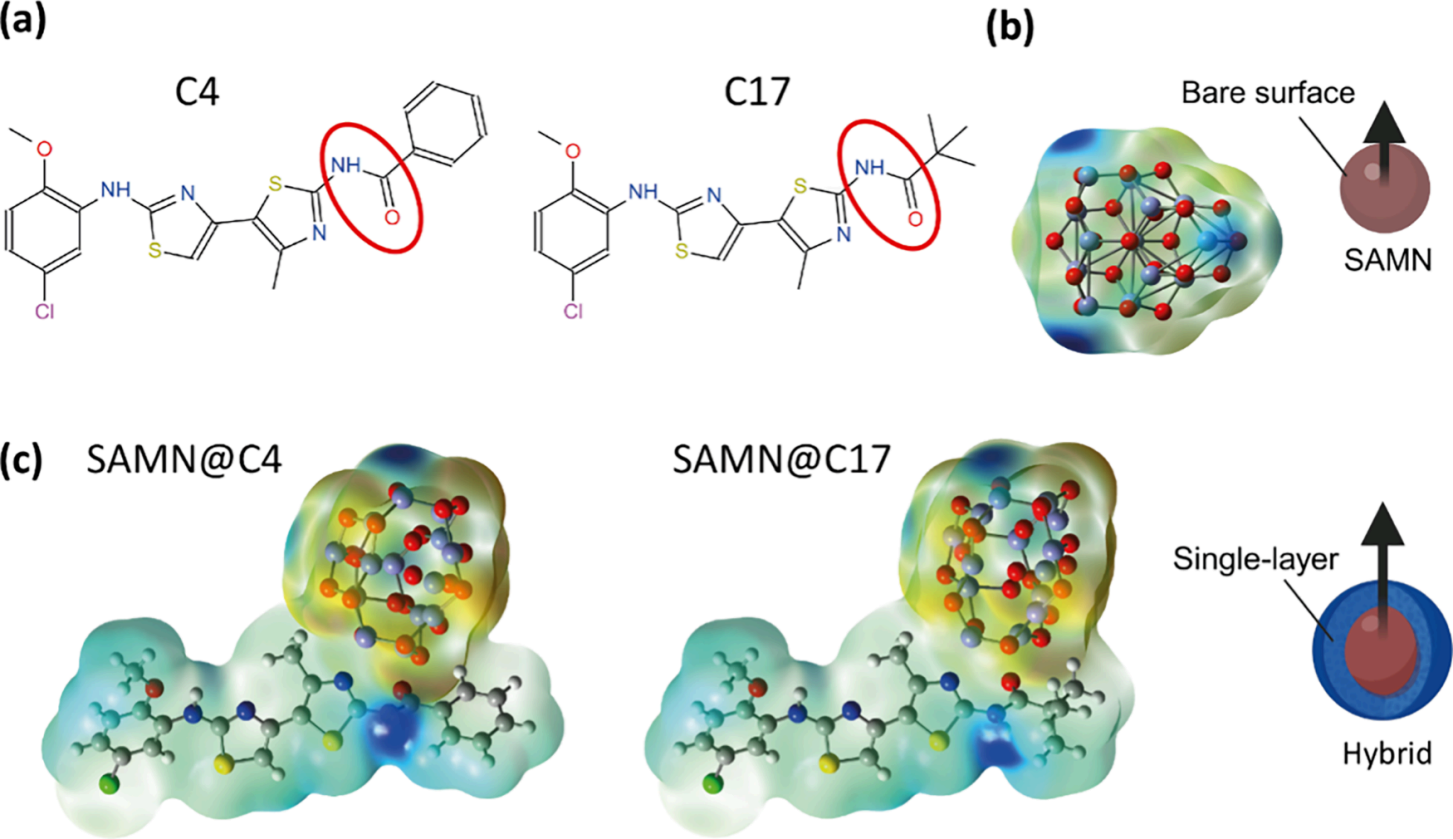
Structures of C4 and C17 and their binding onto SAMNs. (a) Lewis structures of therapeutic molecules with the iron­(III)-amide chelation functionality highlighted in red. Optimized structures of the isolated (Fe_2_O_3_)_7_ cluster (b) and the complexes of C4 and C17 with the ferromagnetic state of the iron oxide cluster (c). The electrostatic potential mapped on the isoelectronic density surface (isovalue 0.0004) is shown, where red or blue areas correspond to negative or positive values, respectively. The arrows in the pictorial representations of bare and surface-modified SAMNs represent the overall dipole moment of the structures.

**Figure 4:**
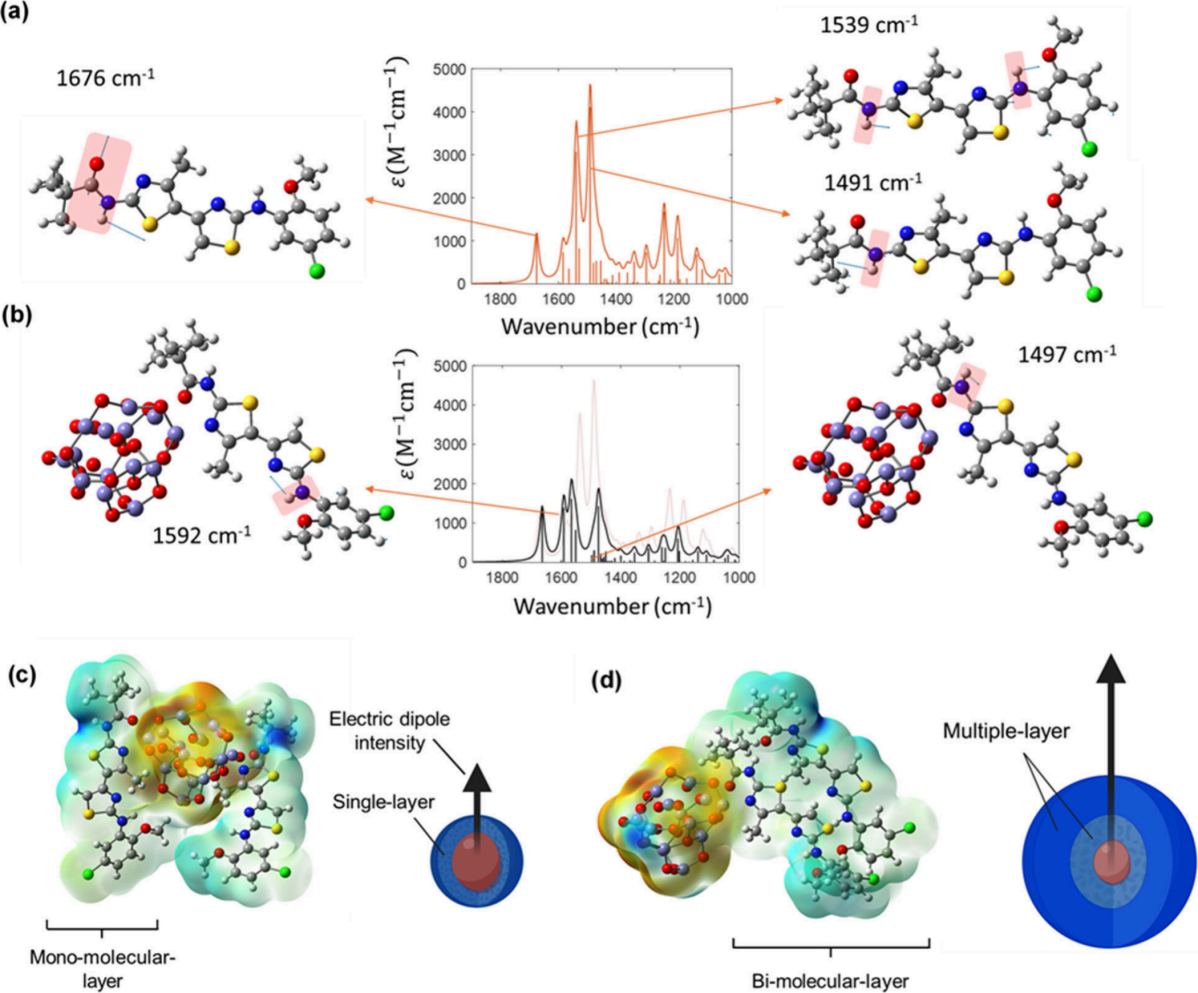
Calculated IR spectra of (a) free C17 molecule in implicit solvent and (b) C17 in the ferromagnetic complex. All frequencies were scaled by the same factor (0.95). Blue arrows on atoms depict normal mode displacement vectors associated with the spectral signal. Optimized structures of complexes comprise the nanoparticle and two C17 molecules. In (c) and (d) the electrostatic potential mapped on the isoelectronic density surface (isovalue 0.0004) is shown, where red or blue colors correspond to negative or positive values, respectively. The arrows represent the overall dipole moment of the structure.

Multilayer formation was also investigated via computational modeling; Figure 4(c,d) shows a propagation of the electronic polarization across layers, as indicated by the magnitude increase in the overall dipole moment of the multilayer complex with respect to the single layer.

Overall, computational modeling highlighted that the induced electronic redistribution results in a modification of the correctors' electrostatic potential, which propagates through the layers of the organic shell, resulting in a overall nonzero charged hybrid surfaces. It can be speculated that the observed preponderance of C17, in terms of proclivity to interact with the aqueous milieu, is related to a higher multilayer dipole in comparison to that of C4. SAMN@C17 zeta potential is high enough to grant sufficient colloidal stability for application purposes. Hence, SAMN@C17 was considered a good candidate for biomedical applications and, therefore, was taken into consideration for the following *in vitro* tests.

To check if the bound corrector retains its activity, SAMN@C17 nanocarrier and naked SAMNs as a control were tested on human embryonic kidney 293 cells (HEK293) (Figure 5(a)) stably expressing a mutated α-sarcoglycan (V247M). This cell model of α-sarcoglycanopathy was used as a validated reference system to verify the efficacy of C17 in recovering a folding defective α-SG mutant.^38^ Nanohybrids were added to the culture media at different concentrations, and cells were incubated for 24 h, according to the scheme of Figure 5(b). Then, the content of the mutated α-SG was analyzed by Western blot on total protein lysates, and glyceraldehyde 3-phosphate dehydrogenase (GAPDH) was used as loading control. It should be considered that, as a folding-defective protein, α-SG is recognized by the endoplasmic reticulum-QC (i.e., quality control system) and degraded through the ubiquitin–proteasome system. Thus, an increase of its cellular levels mirrors the successful rescue of its tertiary structure. A representative Western blot is reported in Figure 5(c). Results show that SAMN@C17 promoted an increase in the protein expression level at all tested concentrations, even though a statistically significant rescue was observed only at the two highest concentrations (75 and 38 μg mL^–1^), as determined by the densitometric analysis shown in Figure 5(d). SAMNs and SAMN@C17 appeared well tolerated by the cells, as no sign of cellular toxicity was registered after 24 and 48 hours of incubation.

**Figure 5:**
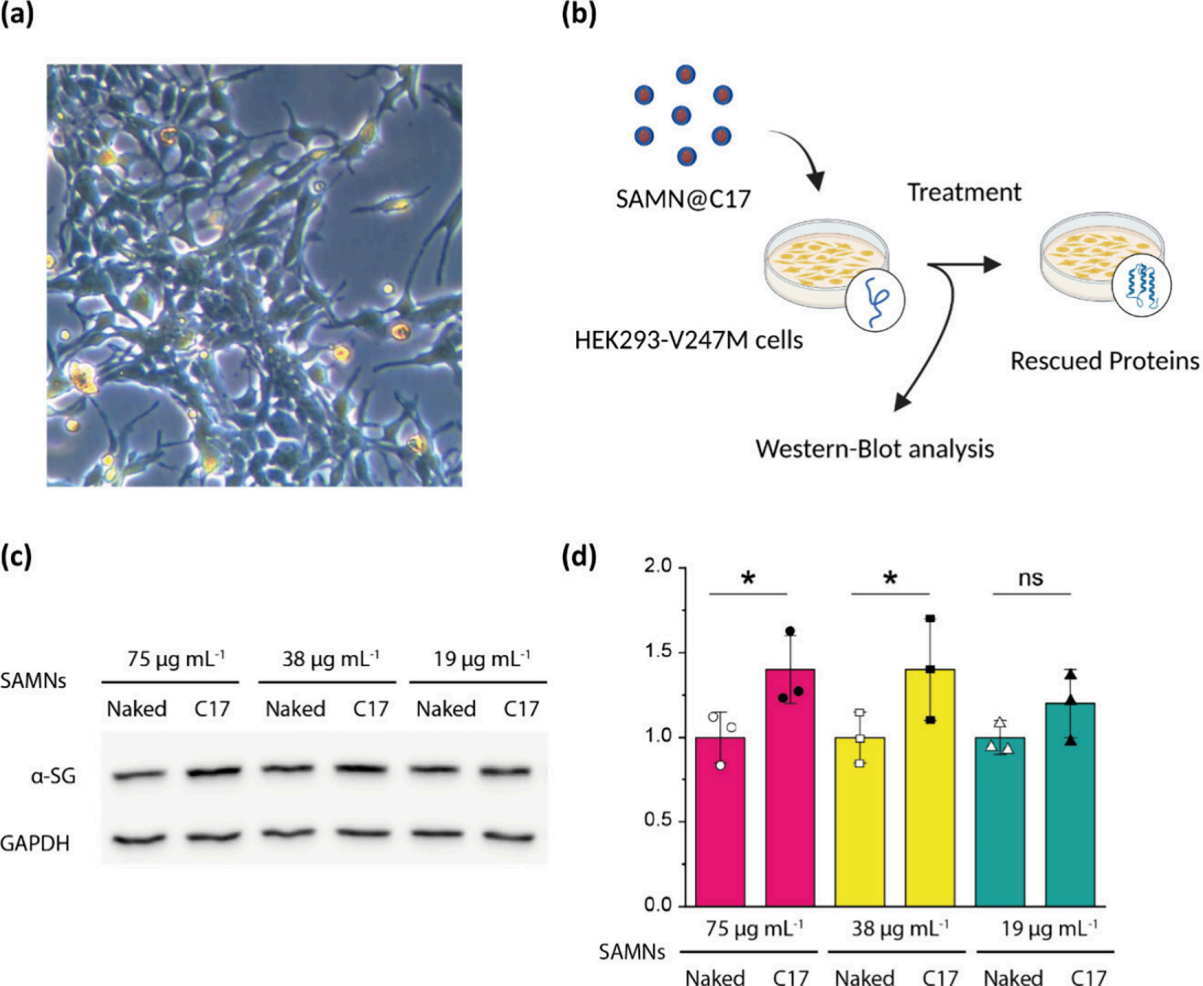
*In vitro* evaluation of SAMN@C17 efficiency in restoring misfolded protein. (a) Phase-contrast optical microscopy image of HEK293-cells. (b) Schematic representation of the experimental workflow showing HEK293 cells, expressing misfolded α-sarcoglycan V247M protein, treated for 24 h with nanoparticles conjugated to C17, followed by Western blot analysis, which confirmed the rescuing of the α-SG protein. (c) Western blot analysis of HEK293-V247M cells treated with SAMN@C17 or naked SAMNs at the indicated concentrations. (d) Quantification of α-SG content by densitometric analysis of Western blot. The average amount of α-SG (±SD) is expressed as fold increase of the protein content with respect to the control (naked SAMNs). Statistical analysis was performed by the one-way ANOVA test followed by Sidak’s multiple comparisons test; n.s., *P* > 0.05; *, *P* < 0.05.

Although C17 remains firmly bound to the iron oxide nanoparticles in aqueous solution, our data suggest that once inside the cells, the compound is released and still functionally active, being able to rescue the folding-defective protein. It is plausible to hypothesize that the molecule is displaced during cellular entry through interactions between the nanocarrier and the hydrophobic compartments of the cell. Even though the precise release mechanism remains to be clarified, these findings are consistent with observations for other molecules (such as peptides and epigallocatechin gallate) that, despite being strongly bound to the carrier, were able to efficiently exert their biological activity once internalized by the cells.^26,27^

Materials generated by the hybridization of nanoparticles with functional molecules are characterized by emerging properties, which can sensibly differ from those of the parent components, as in the present case. The development of a self-assembly wet protocol for the direct and stable immobilization of C4 and C17 CFTR correctors onto SAMNs was presented, demonstrating that an optimal DMSO–water ratio favors thermodynamically the interaction, resulting in the generation of a core–shell system. Hybrid nanomaterials, SAMN@C4 and SAMN@C17, were produced as a result of such a solvent-promoted self-assembly, and they were characterized by a high drug-loading, long-term chemical stability of the therapeutic cargo, well-preserved magnetic moment, and coating robustness. Despite the hydrophobicity of the organic canopies, both core–shell hybrids displayed a zeta potential that differs from zero at physiological pH, and, in particular, nanoimmobilization endowed the C17 nanocarrier with a significantly higher colloidal stability, which is mandatory for a drug carrier.

Moreover, the combination of SAMN@C17 with the intrinsic optical and magnetic properties of the SAMN core resulted in a dual imaging system for cell tracking, making SAMN@C17 a candidate for nanomedicine applications.

The biological activity of SAMN@C17 was observed in HEK293 cells expressing mutated α-SG, showing an increase in the protein level displaying no sign of cell toxicity. These preliminary *in vitro* results lay the basis for further in-depth investigation on the potential of SAMNs as a tool for corrector delivery. Research on a murine animal model for cystic fibrosis or sarcoglycanopathy could be used to study biodistribution, pharmacokinetics, toxicity, and therapeutic effects of SAMN@C17 *in vivo.*

The present contribution enriches the scenario of alternative nanomaterials and immobilization approaches for the next generation of nanomedicine and nanodevices.^39,40^ In addition, SAMNs represent a leap forward in the therapeutic strategy not only for cystic fibrosis but also for other diseases related to protein misfolding.

## Supplementary Material







## Notes

Materials and methods, supplementary results, Tables S1 and S2, and Figures S1, S2, S3, S4, S5, S6, and S7 (PDF)
Video V1: tridimensional rendering of monomolecular complex SAMN@C17 (MP4)
Video V2: tridimensional rendering of bimolecular complex SAMN@C17 (MP4)

## ABBREVIATIONS

SAMNs: Surface Active Maghemite Nanoparticles
CF: Cystic Fibrosis
CFTR: Cystic Fibrosis Transmembrane Conductance Regulator
SERCA1: Sarco-Endoplasmic Reticulum Calcium ATPase 1
DMSO: Dimethyl Sulfoxide
TEM: Transmission Electron Microscopy
TGA: Thermogravimetric Analysis
SQUID: Superconducting Quantum Interference Device
DLS: Dynamic Light Scattering
UV–vis: Ultraviolet–Visible Spectroscopy
FTIR: Fourier Transform Infrared Spectroscopy
DFT: Density Functional Theory
IR: Infrared
ERAD: Endoplasmic Reticulum-associated Degradation
SG: Sarcoglycan
C4: N-[2-(5-chloro-2-methoxy-phenylamino)-4′-methyl-[4,5′]­bithiazolyl-2′-yl]­benzamide
C17: N-(2-(5-chloro-2-methoxyphenylamino)-4′-methyl-4,5′-bithiazol-2′-yl)­pivalamide
NMR: Nuclear Magnetic Resonance
HEK293: Human Embryonic Kidney 293
GAPDH: Glyceraldehyde 3-phosphate dehydrogenase
