# Supplementary material for: Nanohybridization as a Route to a Water-Friendly Therapeutic Tool for Rescuing Misfolded Proteins
Source: ACS Nanosci Au. 2025 Nov 7;6(2):191–200. doi: 10.1021/acsnanoscienceau.5c00119 (PMC13087967; doi:10.1021/acsnanoscienceau.5c00119)
Supplement: Supplementary file 1 [file ng5c00119_si_001.pdf]

Nano-hybridization as a Route to a Water-friendly Therapeutic Tool for Rescuing Misfolded Proteins

Mary Bortoluzzi[a], Aura Cencini[a], Lavinia Rutigliano[b], Graziano Rilievo[a], Alessandro Cecconello[a,c], Federica Tonolo[a], Simone Molinari[d], Roberta Sacchetto[a], Marcello Carotti[e], Dorianna Sandonà[e], Tiziana Martinello[f], Juri Ugolotti[g], Barbara Fresch[h], Lucio Litti[h], Fabio Vianello[a] and Massimiliano Magro[a*].

[a] *Department of Comparative Biomedicine and Food Science, University of Padua, Viale dell'Università 16, 35020 Legnaro (PD), Italy*

[b] *Department of Molecular Medicine, Laboratory affiliated to Istituto Pasteur Italia, Fondazione Cenci Bolognetti, Sapienza, University of Rome, Viale Regina Elena 291, 00161 Rome, Italy*

[c] *Department of Molecular and Translational Medicine, University of Brescia, Viale Europa 11, 25123, Brescia (BS), Italy*

[d] *Department of Geosciences, University of Padua, Via Gradenigo 6, 35131 Padova (PD), Italy*

[e] *Department of Biomedical Sciences, University of Padua, Via U. Bassi 58/b, 35131 Padova (PD), Italy*

[f] *Department of Veterinary Medicine, University of Bari, Piazza Umberto I 1, 70121 Bari (BA), Italy*

[g] *Regional Centre of Advanced Technologies and Materials, Department of Physical Chemistry, Palacky University in Olomouc, Slechtitelu 27, 783 71 Olomouc, Czech Republic*

[h] *Department of Chemical Sciences, University of Padua, Via F. Marzolo 1, 35131 Padova (PD), Italy*

**E-mail: massimiliano.magro@unipd.it; Tel.: 0039-049-8276863*

KEYWORDS: Magnetic nanocarriers, Theragnostic agents, Degenerative disorders, Nano-hybrids, Molecular correctors

1. MATERIALS AND METHODS

1.1 Chemicals

Chemicals were purchased at the highest commercially available purity and were used without further treatment. Fe(III) chloride hexahydrate (97%), sodium borohydride ($NaBH_4$), and ammonia solution (35% in water) were obtained from Merck KGaA (Germany). A series of Nd-Fe-B magnets (N35, 263–287 kJ/m^3 BH, 1170–1210 mT flux density by Powermagnet - Germany) was used for the nanoparticles driving. Detailed description of the synthesis and the physical and chemical characterization of SAMNs are described elsewhere.[1] C4 and C17 compounds were purchased from Rosstek Limited (Paralimni, Cyprus).

1.2 Instrumentation

TGA (thermogravimetric analysis) measurements were collected using an STA 449 C Jupiter (Netzsch Instrument, USA), coupled with QMS 403 Aeolos mass spectrometer (Netzsch Instrument, USA) with a heating rate of 10°C/min, up to 1000°C/min in synthetic air (flow of 70 mL min-1) using N_2 (flow rate of 10 mL/min) as protective gas. FT-IR measurements were performed by a Thermo Nicolet Nexus 670 instrument. TEM (transmission electron microscopy) images were obtained using a JEOL 2010 microscope, operating at 200 kV with a point-to-point resolution of 1.9 Å, and analyzed using Fiji (ImageJ, version 2.9.0). Dynamic light scattering (DLS) and zeta-potential measurements of drug nanocarriers and bare nanoparticles were performed using a Zetasizer instrument (Malvern Instruments LTD, UK). Measurements were performed with three replicates for each sample, at 25°C ± 2°C, with an angle of detection in backscatter. UV-Vis spectra were obtained using a Cary 60 spectrophotometer (Agilent Technologies, USA), in a 1 cm quartz cuvette in the 200 - 800 nm range. Magnetization measurements were collected with a superconducting quantum interference device (SQUID, MPMS XL-7, Quantum Design, USA). Hysteresis loops were recorded at 5 K and 300 K in

the presence of an external magnetic field ranging from −5 to +5 T. The zero-field-cooled (ZFC) and field-cooled (FC) magnetization curve analyses were carried out in the 5 K to 300 K temperature range.

1.3 Cell culture and Immunoblot analysis

HEK293 cells, stably expressing the V247M-α-SG protein (named V247M cells)[2] were maintained in high glucose Dulbecco's Modified Eagle's Medium (DMEM) (Merck KGaA, Darmstadt, Germany) with the addition of 10% FBS (fetal bovine serum purchased from Gibco - Thermo Fisher Scientific, Massachusetts, USA), under a humidified atmosphere containing 5% CO_2 at 37°C.

Treatment with nanoparticles was operated on 105 V247M cells were plated in 12-wells multiwell plates the day before the treatment. The medium was prepared using Merck KGaA, Darmstadt, Germany, at 2X final supplemented with 4% FBS serum. Nanoparticle suspensions were prepared in 50 mL of such medium that was mixed to an equal volume of nanoparticles 0.5 g L^{-1} in water and immediately added to the cells.

Cells with nanoparticle hybrids were incubated for 24h. At the end of the treatments, cells were washed twice with PBS 1X and lysed with 5% sodium deoxycholate (Merck KGaA, Darmstadt, Germany) supplemented with complete protease inhibitor (Merck KGaA, Darmstadt, Germany). Samples were sonicated for 10 minutes on ice using a Fisherbrand™ Model 120 Sonic Dismembrator (Fisher Scientific, Milan, Italy) and then centrifuged at 20000 x g for 30 minutes at 4°C. The supernatants were recovered, and total protein concentration was determined by BCA assay (Thermo Fisher, USA) according to the manufacturer instructions. 30 µg of total cellular proteins were separated by SDS-PAGE and blotted onto a nitrocellulose membrane (Biorad). After 30 min of incubation in blocking solution, membranes were probed with an anti-alpha-SG rabbit monoclonal antibody (Abcam, Italy) (1:2000) and a rabbit polyclonal anti-GAPDH antibody (Merck KGaA,

Darmstadt, Germany) (1:5000) overnight at 4°C. A horseradish peroxidase-conjugate was used as the secondary antibody (Merck KGaA, Darmstadt, Germany), and blots were developed with ECL chemiluminescent substrate (Euroclone).

Chemiluminescence signals were digitally acquired with an Alliance Mini HD9 Imaging System (Uvitec, Cambridge, UK). Band quantification was performed with ImageJ software. The intensities of sarcoglycan bands were normalized using the intensity of GAPDH bands. Values were expressed as means ± standard deviation. Statistical differences among groups were determined by a One-way ANOVA test followed by Sidak multiple comparisons test (GraphPad8 Software, San Diego, CA). A level of confidence of $p < 0.05$ was used for statistical significance.

2. SUPPLEMENTARY RESULTS

2.1. Reaction optimization

The reaction consisted in an overnight incubation of 600 mg L^{-1} SAMN and 200 µM C4 or C17, under agitation at 4°C. 100% DMSO drug solution was poured into SAMN water suspension under vigorous mixing to favor the interaction between the organic and inorganic counterparts. Assessment of cargo loading was carried out using UV-Vis spectroscopy at 324 and 318 nm. Figure 1 (a) shows representative spectra for both correctors where the calculated molar extinction coefficients resulted ε_{324} = 2.6 104 M^{-1} at and ε_{318} = 2.8 104 M^{-1} for C4 and C17, respectively (calibration curves are reported in Figure S1). The amount of bound organic cargo was quantified by measuring the supernatant concentration after the self-assembly reaction and the nanoparticle magnetic removal. The estimated amount of bound correctors was approximately 150 mg g^{-1} SAMNs for both C4 and C17.

Water stability of the nano-immobilized therapeutic cargos was evaluated for the hybrid nano-conjugates (named SAMN@C4 and SAMN@C17) in an aqueous milieu by repeatedly subjecting the

two hybrids to 1 hour water incubations under vigorous agitation, alternated by magnetic separation. The outcome of the stability tests was monitored by UV-Vis spectroscopy. Not surprisingly, drug leakage from SAMNs surface was undetectable. Conversely, pure DMSO led to the complete release of the cargo.

The stability of transported C4 and C17 bound to SAMNs was determined after 14 days of storage at 4°C in water. After this period, bound correctors were released in 100% DMSO and checked by UV-Vis spectroscopy. According to the evaluation, the organic molecules were well-preserved both in terms of spectral profile and absorbance intensity, with residual UV-Vis intensities above 80% and 95% for SAMN@C4 and SAMN@C17, respectively. In order to further validate the formation of the core-shell hybrids, as well as to quantify the drug loading, the optimized reaction products were subjected to an in-depth chemical-physical investigation. Thus, SAMN@C4 and SAMN@C17 were ~~firstly~~ characterized using a superconducting quantum interference device. From the hysteresis loops obtained at 5 K, it is evident that SAMN@C4 and SAMN@C17 showed quantifiable coercivity and remanence, indicating that the systems are in a blocking state below the transition temperature, compatible with the presence of the iron oxide core, as shown in **Figure 1** (b), main text. Remanence and coercivity at 300 K were significantly lower in comparison to the values measured at 5 K, indicating a superparamagnetic state of the samples (the spins of all magnetic nanoparticles oscillate between the orientations of the magnetization axis). Moreover, the magnetic properties of SAMN@C4 and SAMN@C17 nanocarriers were also very favorable for their use as NMR contrast agents, as shown by field-dependent magnetization measured at 300 K (**Figure 1** (b), main text). In comparison to pristine maghemite nanoparticles, the maximum magnetization resulted in a reduction from 71.4 A m^2 kg^{-1} to around 50 A m^2 kg^{-1}. Considering the organic shell diamagnetic nature for C4 and C17, this magnetic characterization enabled quantification of the organic phase bound to the maghemite surface. According to SQUID measurements, the contribution of the diamagnetic (organic) phase to the overall hybrids was around 29% w/w. In **Figure 1** (c), main text, the

superparamagnetic behavior was confirmed by the zero-field cooled (ZFC) and field cooled (FC) magnetization measurements. When the temperature was lowered, the spins of all the magnetic nanoparticles subsequently froze in the magnetically blocked regime. The temperature range where the transition to the magnetically blocked state takes place is documented by a maximum at the ZFC magnetization curve, and the blocking temperature (T_b) can be depicted, where T_b is the temperature at which the average size of nanoparticles is magnetically blocked. In the SAMN@C4 and SAMN@C17 hybrids, the blocking temperatures were 68.0 K and 73.4 K.

The amount of loaded drug was further estimated by thermal gravimetric analysis (TGA). In Figure 1 (d), main text, TGA measurements showed significant losses at approximately 240°C and 400°C. When compared to the reported residual masses, after the thermal treatment, both SAMN@C4 and SAMN@C17 displayed quite comparable and remarkably bulk drug cargos, namely 167.2 mg g^{-1} and 193.5 mg g^{-1} for SAMN@C4 and SAMN@C17, respectively.

2.2. Computational modelling

The cluster $(Fe_2O_3)_7$ was selected as a model for the maghemite nanoparticle surface. The compact ground state structure was already reported[3] and it was used as initial geometry and further optimized at the HSE06[4] level of theory, which was shown to be a good functional hybrid for modeling iron oxide and the adsorption of organic molecules on transition metal surfaces.[5,6] All structures were optimized in vacuum without symmetry constraints. The LANL2DZ basis set with the corresponding effective core potential[7] was used for Fe atoms, while the 6-31 G(d) basis set was employed for all other atoms. Frequency calculations show no imaginary components, confirming the obtained geometries as a minimum of the potential energy surface and providing free energies and the calculated IR spectra. Binding energies (BE) were calculated based on the free energies of the complex, the bare cluster, and the isolated corrector in their respective relaxed geometry. The effect of the solvent was introduced implicitly (with the SMD model[8]) only for charged species. All

calculations were carried out with Gaussian16.[9] In order to model different conditions possibly occurring in the real system, we converged a singlet state in restricted and unrestricted configuration, the open shell ferromagnetic configuration of the neutral cluster, and a highly charged (+6) cluster in a singlet spin state. Different states of the clusters do not change the qualitative picture of the binding with the corrector molecule C17, therefore the binding with C4 was studied only with the more stable neutral-ferromagnetic state of the iron-oxide cluster. **Table S1** summarizes the optimized corrector molecules and clusters with the corresponding energies, while **Table S2** reports the details of the studied complexes. The systems explicitly discussed in the main text are those that resulted being more stable, namely the ferromagnetic configuration of the cluster and the complex with correctors in the isomer 2 form, which are shaded in gray in the Tables.

The electrostatic potential (ESP) mapped on an electron-density iso-surface of the bare neutral iron oxide clusters (**Figure 3** (a), in the main text, red and blue spheres represent oxygen and iron atoms, respectively), shows positive regions (dark blue areas) corresponding to peripherical Fe atoms, identified as preferential binding sites for the corrector molecules. Since C4 and C17 amide I is the putative binding group (*vide supra*), two stable conformers differing in the orientation of the adjacent heterocycle were considered (shown in **Figure S4** for C17). Conformers where the carbonyl is in cis position in respect to the sulfur atom of the heterocycle are lower in energy (free energy difference equals ca. 8 kcal mol^{-1}), for the isolated C4 and C17. However, when the carbonyl is in cis position in respect to the nitrogen atom of the heterocycle, such conformers show a more delocalized negative ESP in correspondence of the amide group, implying a stronger binding affinity with the iron-oxide for both C4 and C17. In this case, for the O-N configuration, the calculated binding free energy (BE) is -41 kcal mol^{-1} against -34 kcal mol^{-1} for the binding of the O-S configuration.

Within the organic-inorganic hybrid, the ferromagnetic spin configuration of the iron oxide cluster was found to be more stable than the non-magnetic configuration, being characterized by an enhanced

internal polarization of the charge distribution compared to the restricted and unrestricted singlet state.

Several binding configurations were obtained as reported in **Figure S5**. Among these, the most notable ones are a chelating configuration where the same Fe atom interacts with the carbonyl and the nitrogen, and a bridging configuration where the corrector C17 interacts with two adjacent metallic centers. Atomic charge analyses revealed that, in the neutral complexes, charge transfer between the iron-oxide cluster and the corrector is minimal. Nonetheless, a non-negligible internal reorganization of electron density is evidenced by the dipole moment, which increases in magnitude in the complex. Indeed, while the isolated correctors have a dipole moment of 1.3 D (C4) and 2.0 D (C17) and the ferromagnetic cluster has a dipole moment of 6.3 D (arrows in **Figure 4** (c), main text), the magnitude of the electric dipole moment increases to 18.4 D (C4) and 18.6 D (C17) in the cluster-corrector complexes as shown in **Figure 4** (d) (main text) for C17.

In **Figure 4** (d), main text, the effect of the electronic redistribution of the electrostatic potential of the complex can be observed, where a positive ESP is found around the corrector in correspondence of the binding site.

To further detail the organic-inorganic component interactions in the nano-complex, simulated IR spectra were obtained employing normal mode analysis for all the ground state structures. First, C17 free molecule spectrum was calculated, and the region of interest is reported in Figure 6(a), main-text, confirming the assignment of the experimental reference peak at around 1676 cm^{-1} dominated by the CO stretching, and the articulated amide II band around 1500 cm^{-1}, where several modes coupling in-plane NH bending with CN stretching can be identified. IR spectra of the individual corrector molecules, including the entire spectra of C4 and C17 are reported in **Figure S6**.

Calculated spectra of the organic-inorganic complexes were then obtained. The IR spectrum of SAMN@C17 complex shows a loss of intensity in the amide II region, as shown in **Figure 4**(b), main text, similarly to the experimental spectra. It should be noticed that the residual signal (i.e., the peak

at 1592 cm^{-1}) is mainly due to normal modes involving the in-plane bending of the secondary amine, not involved in the binding of the iron-oxide surface. On the other hand, the signal due to the amide involved in the binding, i.e., the peak at 1497 cm^{-1}, is reduced in all the complexes, confirming its engagement in a strong interaction with the nanoparticle.

Table S1 Summary of correctors and bare clusters studied through computational method with corresponding electronic energy (E) and Gibbs free energy (G). Relevant energy differences are reported in the Δ column.

**TABLE 1**	notes	E(Hartree)	G(Hartree)	Δ(kcal/mol)	notes
**C17**					
isomer 1	vacuum	-2361.11887	-2360.79781		
isomer 2	vacuum	-2361.10349	-2360.78517	7.9	ΔG w.r.t. isomer 1
isomer 2	solvent	-2361.12760	-2360.80691		
**C4**					
isomer 1	vacuum	-2434.84321	-2434.55412		
isomer 2	vacuum	-2434.82857	-2434.54100	8.2	ΔG w.r.t isomer 1
isomer 2	solvent	-2434.85509	-2434.56710		
**BARE CLUSTERS**					
1-neutral singlet	Restricted	-3305.41869	-3305.38921		
2-neutral singlet	Unrestricted	-3305.66061			
3-neutral ferromagnetic	2S+1=71	-3306.42212	-3306.41428	-477.8	ΔE w.r.t unrestricted singlet
4-charged in solvent	6+ cation	-3304.59732	-3304.57415		

Notice that the calculated intensities of the amide I band is not suppressed compared to amide II band as observed in the experimental spectrum. This can be understood because DFT IR intensities are intrinsic as they are proportional to the dipole moment derivatives along the normal mode displacement ($\partial\mu/\partial Q$). The relative intensities in the experimental ATR spectra reflect additional factors not included in the calculation. In particular, the local electromagnetic field at the interface is frequency- and orientation-dependent and the dielectric response of the environment (notably the strong H_2O bending absorption around 1640 cm^{-1}) selectively damps the effective field driving the C=O stretch. These effects can suppress the experimental Amide I band relative to Amide II and they are not included in the DFT predictions.

To investigate the binding of multiple corrector molecules, we studied complexes where two C4 or C17 molecules interact with the same iron oxide cluster. In one structure, two C17 correctors interact directly with the surface, **Figure 4**(c), main text, while in a different complex, **Figure 4**(d), main text, the second molecule binds to the first corrector through the electrostatic interaction between the amide group and the positive electrostatic potential induced by the first binding event (see also **videos V1 and V2**, for animated tridimensional representations of monomolecular and bimolecular complexes). The same layered bonding was investigated for C4 (**Figure S7**). The binding energy is stronger for direct interaction (-31.4 kcal mol^{-1}) but is significant also for the interaction mediated by the polarization of the first binder (-7 kcal mol^{-1} for C4 and -4.1 kcal mol^{-1} for C17). The study of the electrostatic potential mapped on the isodensity surface confirms the trend of charge polarization, where positive ESP values characterize the organic molecule vicinity. The dipole moment of the double-layer complex increases to 23.7 D for C4 and to 25.1 D for C17, showing that the polarizing capability of the iron-oxide surface extends beyond directly bound molecules.

Table S2 Summary of corrector-cluster complexes studied computationally with corresponding electronic energy and Gibbs free energy. The energy differences refer to the free energy of binding.

**TABLE 2**	**complex C17-isomer 2**		ΔG (kcal/mol)	notes	**Complex C17-isomer 1**		ΔG (kcal/mol)
1-neutral singlet	-5666.61826	-5666.24187	-42.4				
3-neutral ferromagnetic	-5667.61837	-5667.26471	-40.9		-5667.60377	-5667.25341	-33.9
4-charged in solvent	-5665.78046	-5665.40221	-13.3				
complex neutral ferromagnetic+2 x C17	-8028.75493<br>-8028.80138	-8028.05642<br>-8028.09994	-4.1<br>-31.4	stacking<br>direct			
	**complex C4-isomer 2**				**complex C4-isomer 1**		
3-neutral ferromagnetic	-5741.34312	-5741.02168	-41.7		-5741.33102	-5741.00960	-25.9
complex neutral ferromagnetic+2 x C17	-8176.20330	-8175.57451	-7.4	stacking			

3. SUPPLEMENTARY FIGURES

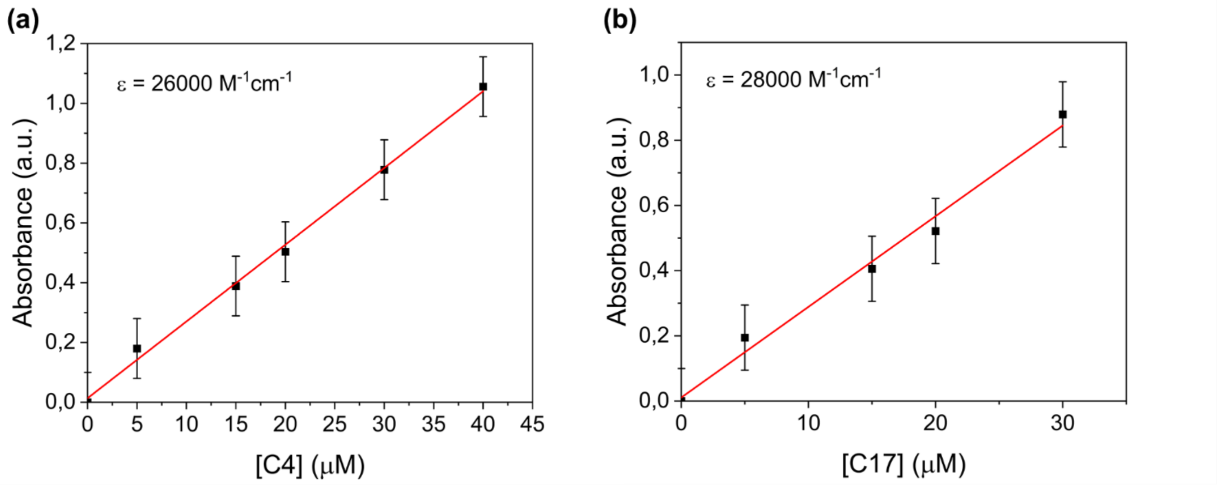

Figure S1 Calibration curves of C4 (a) and C17 (b) according to the Lambert Beer Law.

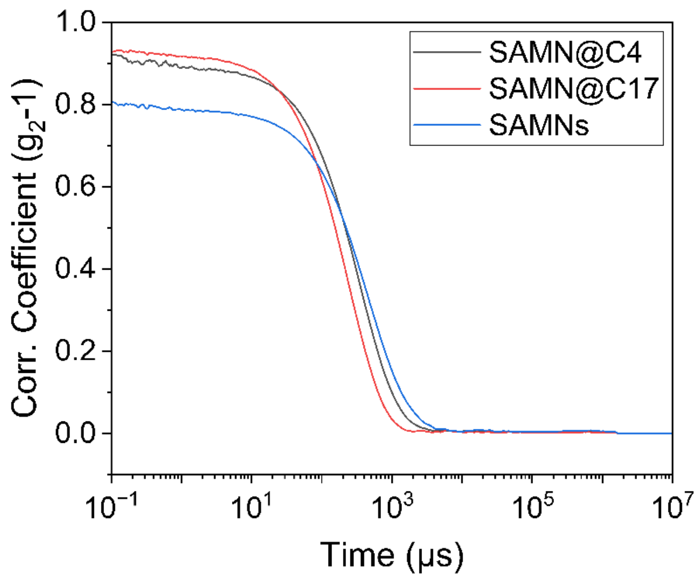

Figure S2 Representative z-size correlograms (i.e., plots of the intensity auto-correlation coefficients (g2-1) as a function of correlation time) of z-size measurements of C4 (black line), C17 (red line) and bare SAMNs (blue line).

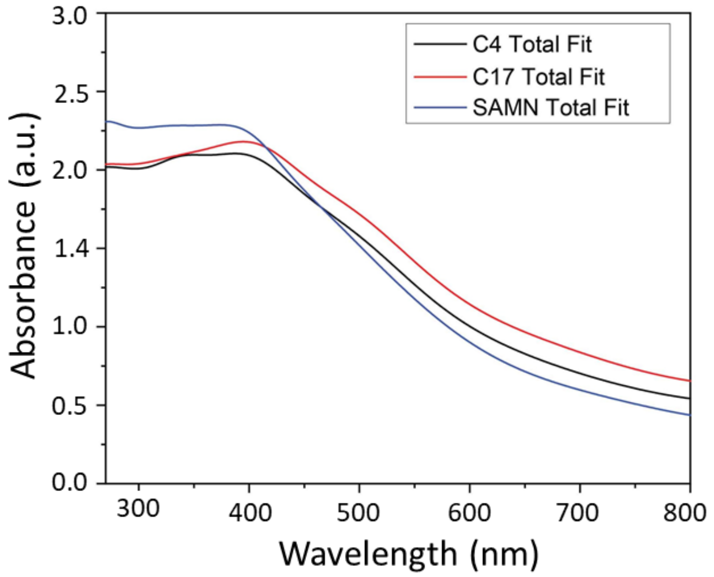

Figure S3 Simulation of SAMNs, SAMN@C4, SAMN@C17 UV-Vis spectra.

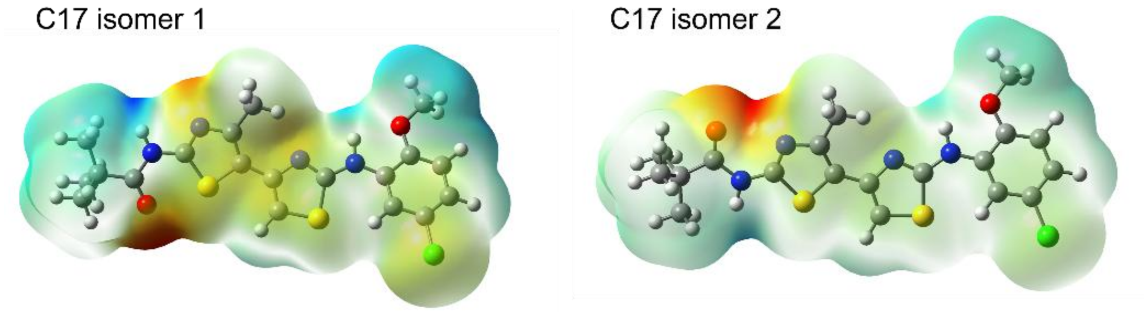

Figure S4 Optimized structure and the electrostatic potential mapped on the isoelectronic density surface (isovalue 0.0004) of the two isomers of the corrector C17 molecules.

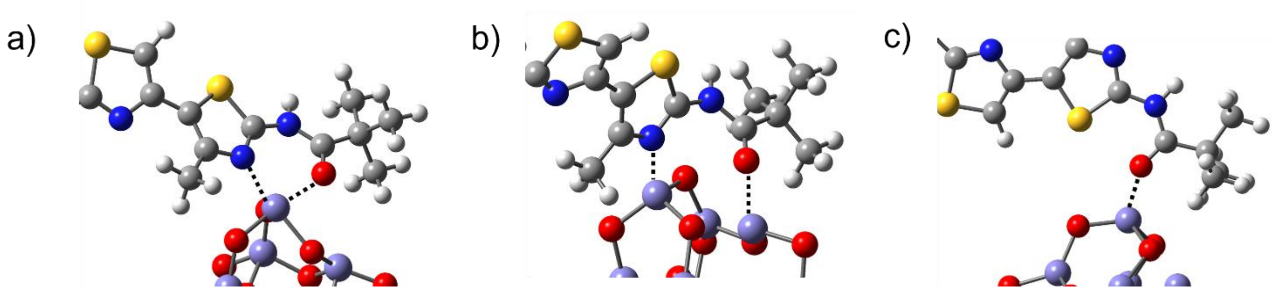

Figure S5 Optimized geometries of different binding sites obtained for C17 interacting with non-magnetic, magnetic, and charged clusters, all representative of different possible configurations for the binding to the nanoparticle. A chelating configuration was found where the same Fe atom interacts with the O-N pair in the non-magnetic and charged cluster (a), a bridging configuration, where the O-N pair interacts with two adjacent metallic centers, is detected in the optimized geometry of the magnetic cluster (b), on top binding was found for the O-S conformer (isomer 1 of C17) (c).

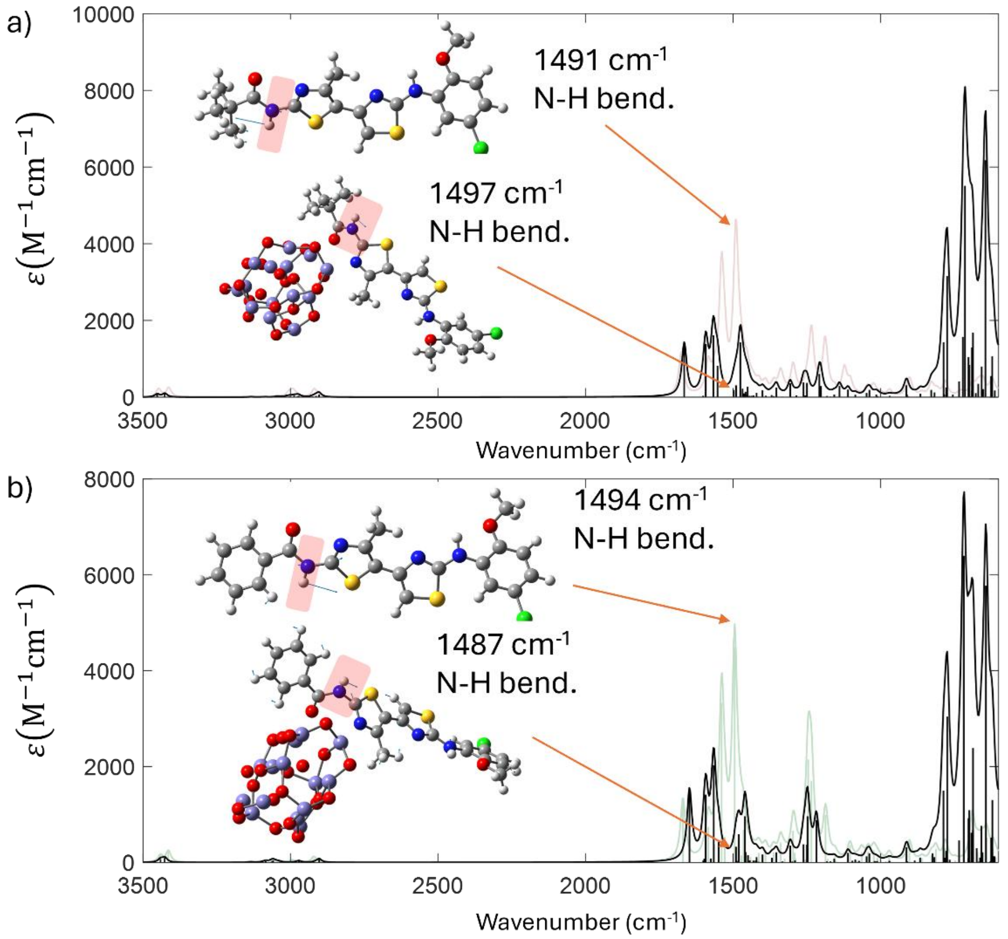

Figure S6 Calculated IR spectra of (a) C4 and (b) C17 correctors in implicit solvent (light lines) and in the ferromagnetic complex (black lines). All frequencies were scaled by the same factor (0.95). The insets highlight the normal mode associated to the NH bend whose intensity is suppressed by the engagement of the mojety in the binding with the cluster.

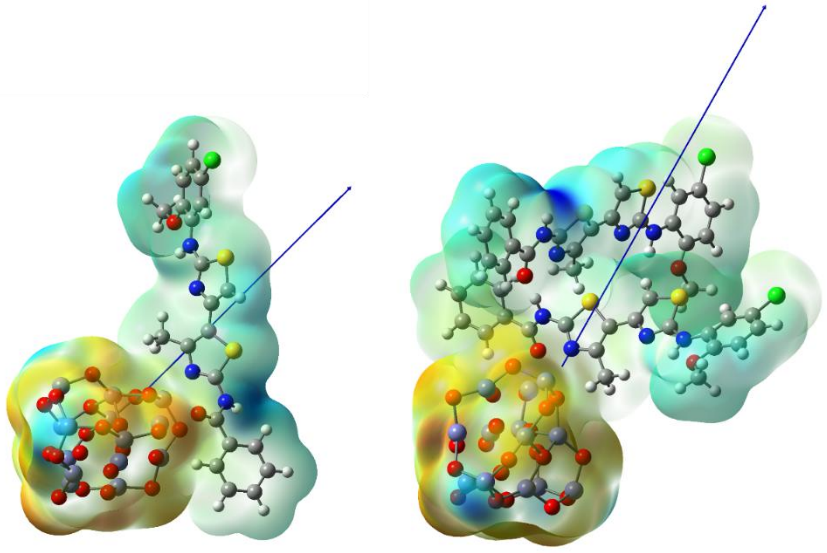

Figure S7 Optimized structures of the complexes between the nanoparticle and one (left) and two (right) C4 molecules. The electrostatic potential mapped on the isoelectronic density surface (isovalue 0.0004) is shown, where red (blue) corresponds to negative (positive) values. The arrows represent the overall dipole moment of the structures.

3. REFERENCES

(1) Magro, M.; Sinigaglia, G.; Nodari, L.; Tucek, J.; Polakova, K.; Marusak, Z.; Cardillo, S.; Salviulo, G.; Russo, U.; Stevanato, R.; Zboril, R.; Vianello, F. Charge Binding of Rhodamine Derivative to OH-Stabilized Nanomaghemite: Universal Nanocarrier for Construction of Magnetofluorescent Biosensors. *Acta Biomater* 2012, *8* (6), 2068–2076. https://doi.org/10.1016/j.actbio.2012.02.005.

(2) Bianchini, E.; Fanin, M.; Mamchaoui, K.; Betto, R.; Sandonà, D. Unveiling the Degradative Route of the V247M α-Sarcoglycan Mutant Responsible for LGMD-2D. *Hum Mol Genet* 2014, *23* (14), 3746–3758. https://doi.org/10.1093/hmg/ddu088.

(3) Erlebach, A.; Hühn, C.; Jana, R.; Sierka, M. Structure and Magnetic Properties of (Fe2O3)n Clusters (n = 1-5). *Physical Chemistry Chemical Physics* 2014, *16* (48), 26421–26426. https://doi.org/10.1039/c4cp02099e.

(4) Heyd, J.; Scuseria, G. E. Efficient Hybrid Density Functional Calculations in Solids: Assessment of the Heyd-Scuseria-Ernzerhof Screened Coulomb Hybrid Functional. *Journal of Chemical Physics* 2004, *121* (3), 1187–1192. https://doi.org/10.1063/1.1760074.

(5) Meng, Y.; Liu, X. W.; Huo, C. F.; Guo, W. P.; Cao, D. B.; Peng, Q.; Dearden, A.; Gonze, X.; Yang, Y.; Wang, J.; Jiao, H.; Li, Y.; Wen, X. D. When Density Functional Approximations Meet Iron Oxides. *J Chem Theory Comput* 2016, *12* (10), 5132–5144. https://doi.org/10.1021/acs.jctc.6b00640.

(6) Alarcón Villaseca, S.; Levchenko, S. V.; Armbrüster, M. CO Adsorption on the GaPd(111) Surface: A Comparative DFT Study Using Different Functionals. *Physical Chemistry Chemical Physics* 2016, *18* (21), 14390–14400. https://doi.org/10.1039/c6cp01820c.

(7) Hay, P. J.; Wadt, W. R. *Ab Initio* Effective Core Potentials for Molecular Calculations. Potentials for the Transition Metal Atoms Sc to Hg. *J Chem Phys* 1985, *82* (1), 270–283. https://doi.org/10.1063/1.448799.

(8) Marenich, A. V.; Cramer, C. J.; Truhlar, D. G. Universal Solvation Model Based on Solute Electron Density and on a Continuum Model of the Solvent Defined by the Bulk Dielectric Constant and Atomic Surface Tensions. *Journal of Physical Chemistry B* 2009, *113* (18), 6378–6396. https://doi.org/10.1021/jp810292n.

(9) Frisch, M. J. T. G. W.; Schlegel, H. B.; Scuseria, G. E.; Robb, M. A.; Cheeseman, J. R.; Scalmani, G.; Barone, V.; Petersson, G. A.; Nakatsuji, H.; Li, X.; Caricato, M.; Marenich, A. V.; Bloino, J.; Janesko, B. G.; Gomperts, R.; Mennucci, B.; Hratchian, H. P.; Ortiz, J. V.; Izmaylov, A. F.; Sonnenberg, J. L.; Williams-Young, D.; Ding, F.; Lipparini, F.; Egidi, F.; Goings, J.; Peng, B.; Petrone, A.; Henderson, T.; Ranasinghe, D.; Zakrzewski, V. G.; Gao, J.; Rega, N.; Zheng, G.; Liang, W.; Hada, M.; Ehara, M.; Toyota, K.; Fukuda, R.; Hasegawa, J.; Ishida, M.; Nakajima, T.; Honda, Y.; Kitao, O.; Nakai, H.; Vreven, T.; Throssell, K.; Montgomery, J. A.; Peralta, J. E.; Ogliaro, F.; Bearpark, M. J.; Heyd, J. J.; Brothers, E. N.; Kudin, K. N.; Staroverov, V. N.; Keith, T. A.; Kobayashi, R.; Normand, J.; Raghavachari, K.; Rendell, A. P.; Burant, J. C.; Iyengar, S. S.; Tomasi, J.; Cossi, M.; Millam, J. M.; Klene, M.; Adamo, C.; Cammi, R.; Ochterski, J. W.; Martin, R. L.; Morokuma, K.; Farkas, O.; Foresman, J. B.; Fox, D. J. Gaussian 16, Revision B.01, Gaussian, Inc. Wallingford CT 2016.
